# The Utility of Low-Dose-Dobutamine Stress Echocardiography in Patients with Heart Failure with Reduced Ejection Fraction: An Update

**DOI:** 10.3390/diagnostics13182920

**Published:** 2023-09-12

**Authors:** Lamprini Tsigkriki, Panagiota Kleitsioti, Fotis Dimitriadis, George Sidiropoulos, Stelina Alkagiet, Dimitris Efstratiou, Maria Kalaitzoglou, Dafni Charisopoulou, Michail Siarkos, Angeliki-Despoina Mavrogianni, Pinelopi Giannakopoulou, John Zarifis, George Koulaouzidis

**Affiliations:** 1 Cardiology Department, General Hospital G. Papanikolaou, 57010 Thessaloniki, Greece; linatsigr@gmail.com (L.T.); pennykleitsioti@yahoo.com (P.K.); ftdimitriadis@gmail.com (F.D.); sidiropoulos.georges@gmail.com (G.S.); salkayed@yahoo.gr (S.A.); dimitref2@gmail.com (D.E.); mariakalaitzo25@gmail.com (M.K.); kosmed@hotmail.com (M.S.); amavrogianni@yahoo.com (A.-D.M.); giannakpop@hotmail.com (P.G.); zarifis.john@gmail.com (J.Z.); 2 Great Ormond Street Hospital, London WC1N 3JH, UK; dafnithess@yahoo.co.uk; 3 Department of Biochemical Sciences, Pomeranian Medical University, 70-204 Szczecin, Poland

**Keywords:** heart failure with reduced ejection fraction (HFrEF), low-dose-dobutamine stress echocardiogram, viability, cardiac resynchronization therapy, aortic stenosis

## Abstract

Despite significant advancements in medical therapy, heart failure with reduced ejection fraction (HFrEF) continues to be a significant cause of death and disability. Reversible ischaemic left ventricular dysfunction due to viable myocardium is one such contributing factor. In these cases, coronary revascularization has shown promise in improving left ventricular function and prognosis. For patients with HFrEF and wide QRS, cardiac resynchronization therapy (CRT) is an effective option to address electromechanical dyssynchrony. However, approximately 30% of patients do not respond positively to CRT, highlighting the need to refine candidate selection for this treatment. In some patients with reduced HFrEF, there is a condition known as classical low-flow, low-gradient aortic stenosis (AS) that may be observed. This condition is characterized by a low transaortic flow, which leads to reductions in both the transaortic mean gradient and aortic valve area. Decision-making regarding revascularization, CRT, and pharmacological treatment play a crucial role in managing HFrEF. Cardiac imaging can be valuable in guiding decision-making processes and assessing the prognosis of patients with HFrEF. Among the imaging modalities, dobutamine stress echocardiography has come a long way in establishing itself as a feasible, safe, effective, relatively cheap non-invasive technique. The aim of this review is to explore the current literature on the utility of low-dose stress echocardiography in diagnosing and prognosticating patients with HFrEF.

## 1. Introduction

Stress echocardiography is a diagnostic method that has broad implications in various medical conditions. It is primarily used for diagnosis and prognosis in patients with coronary artery disease (CAD), as well as assessing myocardial viability. Other indications of stress echocardiography include the quantification of contractile reserve (CR) in cardiomyopathies, the assessment of valvular heart disease and congenital heart disease, and the evaluation of diastolic function and pulmonary hypertension. The main advantages of stress echocardiography are that it is a simple, inexpensive, widely available, and radiation-free method [1,2].

Stress echocardiography involves the dynamic evaluation of myocardial structures, function, and hemodynamic status under physiological or pharmacological stress. The most commonly used stressors in this procedure are dobutamine, dipyridamole, and exercise [3,4]. Dobutamine stress echocardiography (DSE) utilizes a synthetic catecholamine called dobutamine, which primarily stimulates β1 adrenergic receptors and, to a lesser extent, α1 and β2 receptors. At a low dosage, dobutamine enhances coronary blood flow, leading to an improvement in myocardial systolic function [2,3,4].

During the low-dose-dobutamine stress echocardiography (LDDSE) procedure, the patient undergoes a dobutamine stress using standardized incremental infusions of 5, 10, and 20 μg/kg/min. Each infusion dose is administered for a duration of up to five minutes. It is mandatory to acquire images from all available views at the conclusion of each stage. The protocol is considered completed if there is a 10% increase in heart rate. However, in cases where the operator anticipates an excessive rise in heart rate, an additional stage at 15 μg/kg/min may be included [2,3,4,5].

Wall motion evaluation using a 16- or 17-segment model is strongly advised for an accurate assessment. The evaluation involves visually scoring the motion in each assessable segment, employing a four-step scale: one for normal, two for hypokinetic, three for akinetic, and four for dyskinetic. To facilitate serial comparisons, a wall motion score (WMS) can be calculated [2,3,4]. This score is determined by summing up the individual segmental wall motion scores, resulting in the global wall motion score. Additionally, the wall motion score index is obtained by dividing the wall motion score by the total number of segments [4]. A wall motion score index of one signifies normal contraction, whereas a higher score indicates the presence of wall motion abnormalities. For assessing myocardial viability, evaluating wall thickness is valuable. A diastolic wall thickness of ≤5 mm at rest suggests nonviability, and when combined with an absent contractile response to dobutamine, it enhances diagnostic certainty. It is recommended to perform a corresponding assessment of global and regional function during each stage of stress, and comparisons should be made between baseline and stress recordings [2,3,4].

Heart failure (HF) is a heterogeneous disease associated with a poor prognosis and reduced quality of life, despite advancements in pharmacological and interventional treatments [6]. Decision-making regarding revascularization, cardiac resynchronization therapy (CRT), and pharmacological treatment plays a crucial role in managing HF. Assessing prognosis and identifying high-risk patients enables targeted specialist care and appropriate follow-up, aiming to reduce hospitalizations and slow down disease progression. Stress echocardiography can be a valuable tool in guiding decision-making processes and assessing the prognosis of patients with heart failure with reduced ejection fraction (HFrEF).

The aim of this narrative review is to explore the literature of the last 10 years on the utility of low-dose stress echocardiography in diagnosing and prognosticating patients with heart failure with reduced ejection fraction.

## 2. Viability and LDDSE

Despite the considerable progress made in the management of cardiovascular (CV) disease, HF remains a major cause of morbidity and mortality. In fact, two-thirds of patients with HF are affected by CAD, which further exacerbates the condition. Identifying the appropriate candidates for revascularization, in addition to guideline-directed medical therapy, is a crucial challenge in the treatment of patients with HF of ischaemic origin (IHF). Medical therapy remains the cornerstone of treatment for these patients, and determining the potential benefits of revascularization is of utmost importance.

The LDDSE exhibits a considerable level of sensitivity (ranging from 77% to 89%) and specificity (ranging from 68% to 93%) not only during the postinfarction phase [7,8] but also in the chronic phase (with sensitivity at 82% and specificity at 92%) [9]. In a comprehensive metanalysis conducted by Romero et al., it was revealed that cardiac magnetic resonance (CMR) employing a low dose of dobutamine demonstrated excellent sensitivity (81%) and specificity (91%) for detecting myocardial viability [10].

In their study, Li et al. included a cohort of 30 patients who had a previous history of percutaneous coronary intervention (PCI) and an average left ventricular ejection fraction (LVEF) of 32.6 ± 15.4 [11]. These patients underwent contrast echocardiography with and without LDDSE. The combined utilization of contrast echo and LDDSE not only demonstrated an increased sensitivity and specificity in detecting viable myocardium but also resulted in an improved ejection fraction (EF) following PCI.

On top of this study, another study was conducted by Ghaffar et al., in which they tried to determine if the extent of the viable myocardium in patients with left ventricle failure could predict short- and long-term advantages after coronary artery bypass grafting (CABG) compared with medical therapy [12]. In that study, 250 patients with ≥4 resting segment abnormalities during a rest echocardiogram with a mean EF = 32 ± 7 underwent LDDSE for an assessment of myocardial viability. Half of the patients had undergone CABG and half treated medically. Each WMS was calculated during a low-dose dobutamine infusion. Based on the WMS, the patients were categorized into groups with extensive (WMS < 2.00), intermediate (WMS = 2.00–2.49), and limited (WMS ≥ 2.50) viability. In patients with extensive viability, after a 2-year follow-up, survival was better among patients after revascularization. Among patients with intermediate viability, it was observed a statistically significant early improvement in survival in the revascularized group. Finally, in the limited viability group, revascularized patients had worse short-term survival compared with the medically treated patients. Summarizing, patients with extensive and intermediate viability seem to benefit from CABG, but patients with limited viability have poorer short-term outcome.

Panza et al. investigated the role of myocardial viability in identifying patients with ischaemic cardiomyopathy who might benefit from surgical revascularization [13]. The study group consisted of 601 patients from the STICH trial, with LVEF ≤ 35%, who had a myocardial viability assessment with single-photon-emission computed tomography or LDDSE. Eighty-one percent (81%) of the study group were considered to have myocardial viability and 19% to not have viability. The study findings revealed that the presence of viable myocardium was significantly associated with improvements in left ventricular systolic function, regardless of the treatment received. However, it is important to note that this improvement in systolic function was not found to be correlated with long-term survival.

## 3. Viability with Strain and Strain Rate during LDDSE

During the last years, new echocardiographic quantitative modalities have been used in assessing myocardial performance. Strain and strain rate have derived from a high-frame-rate tissue doppler imaging (TDI) and due to its angle independency is a more accurate way of quantifying regional myocardial deformation, both at rest and during stress [14].

Initially, Ran et al. enrolled 36 patients with a mean EF = 40 ± 6, who underwent a two-dimensional echo combined with a 2D speckle tracking imaging (STI), at rest and after infusion of adenosine at 140 µg/kg/min over a period of 6 min [15]. Longitudinal, radial, and circumferential strains and strain rate were calculated. After adenosine infusion, longitudinal and radial strains were improved significantly in the viable group, while there was no difference in the circumferential strain. Strain emerged to be more accurate than strain rate in terms of regional myocardial function assessment.

Moreover, Li et al. also aimed to assess the sensitivity and specificity of patients with old myocardial infarction (MI) and LV dysfunction by LDDSE and STI [16]. They studied 33 patients with a mean EF of 43.2 ± 5.7. Longitudinal, circumferential, and radial strains and strain rate were acquired at rest and under stress. It emerged that when LDDSE was combined with STI, the sensitivity and specificity were better than LDDSE alone, while after a multivariable analysis, the longitudinal strain and strain rate were independent predictors of viability.

Wang et al. explored the sensitivity and specificity of delayed-enhancement magnetic resonance imaging (DE-MRI) combined with STE and LDDSE for the detection of viability in the myocardium [17]. In that study, the researchers recruited 35 patients who were hospitalized for MI and had regional wall motion abnormalities, with LVEF < 50%. They aimed to evaluate the efficacy of different tests in detecting myocardial viability. The results showed that delayed-enhancement magnetic resonance imaging (DE-MRI) exhibited a sensitivity, specificity, and accuracy of 92.41%, 89.19%, and 91.32%, respectively, in detecting myocardial viability. In comparison, a parallel test involving two main parameters in STE—longitudinal strain and longitudinal strain rate—demonstrated an improved sensitivity, specificity, and accuracy between baseline and LDDSE (71.72% vs. 91.72%, 70.27% vs. 85.14%, and 71.23% vs. 89.50%, *p* < 0.05). Although the parallel test of STE with LDDSE showed a high sensitivity for detecting viable myocardium, its specificity and accuracy were lower compared to DE-MRI, even when combined with LDDSE. However, the combination of these two methods resulted in a significant improvement in the sensitivity, specificity, and accuracy for the assessment of viable myocardium. Therefore, utilizing both DE-MRI and STE with LDDSE provides a more robust approach for evaluating VM compared to using either method alone.

## 4. Cardiac Resynchronization Therapy and LDDSE

CRT is increasingly used in patients with heart failure. It effectively results in an improvement of cardiac function and clinical status as well as a reduction in morbidity and mortality in appropriately selected individuals [18,19]. However, up to 30% of patients do not respond favourably to CRT [20]. The only established predicting factors for a CRT response are the QRS width and morphology, which, together with LVEF, are the only criteria in the selection of patients. The role of cardiac imaging in identifying responders has been evaluated mostly in observational studies. Contractile reserve, cardiac dyssynchrony, myocardial scar, guidance of LV lead implantation based on imaging have been associated with a response to CRT [18]. Several observational studies have investigated the role of stress echocardiography in identifying CRT responders (Table 1).

The LODO-CRT trial investigated the role of LDDSE in predicting a CRT response (Table 1) [21]. Overall, 77% of participants showed a contractile reserve. In the group with a left ventricle contractile reserve (LVCR), 87% of the patients were found to be CRT responders. In contrast, only 42% in the group without LVCR exhibited an echocardiographic response (*p* < 0.001). LVCR demonstrated a sensitivity of 90% and a positive predictive value of 87% in predicting echocardiographic CRT responders. Furthermore, through a multivariable analysis, LVCR and interventricular dyssynchrony were identified as independent predictors of the CRT response. When both factors were present concurrently, the combination showed a specificity of 99% and a sensitivity of 83% in accurately detecting responders.

In patients diagnosed with dilated cardiomyopathy and left bundle branch block (LBBB), a response to LDDSE may indicate a promising outcome for CRT (Table 1) [22]. Specifically, if the wall motion score index improves by more than 0.7 and the EF increases by at least 14% during the dobutamine infusion at a dose of 20 µg/kg/min, it suggests a positive response to the stress test. In such cases, the implementation of CRT has the potential to lead to a nearly complete restoration of left ventricular systolic function.

The ViaCRT study showed that CR could predict the echocardiographic response to CRT in patients with ischaemic HF and nonischaemic HFrEF (Table 1) [23]. However, when considering the clinical response to CRT, the association with CR was only significant in the nonischaemic group (*p* = 0.03). In the multivariable analysis, it was observed that a preserved CR emerged as the sole independent predictor of a response to CRT in both the ischaemic and nonischaemic groups.

Stankovic et al. aimed to explore the relationship between CR, LV dyssynchrony, and LVEF during an LDDSE, and how these factors contributed to the efficacy of CRT (Table 1) [24]. For these reasons, they employed the following imaging methods: apical rocking (ApRock), LDDSE, and contrast-enhanced magnetic resonance imaging (MRI). Apical rocking is a surrogate marker of dyssynchrony and can be quantified by measuring the apical transverse motion (ATM). Among the patients who positively responded to CRT, a higher proportion (67%) was observed to be women. Additionally, these responders were more likely to have nonischaemic cardiomyopathy and wider QRS complexes compared to nonresponders. The ApRock parameter measured during DSE demonstrated a strong predictive value for the CRT response (*p* < 0.001). Furthermore, ApRock showed an inverse correlation with changes in LVEF (*p* < 0.001). In contrast, changes in LVEF during DSE did not show a significant association with the response to CRT (*p* = 0.082). The researchers conducted a linear regression analysis, which revealed an inverse association between changes in LVEF during DSE and two factors: the total scar burden (*p* < 0.001) and the DSE-induced change in ApRock amplitude (*p* < 0.001). This suggested that patients with a higher scar burden and a smaller DSE-induced change in ApRock amplitude were less likely to experience significant improvements in LVEF during the stress test. Additionally, the study employed a Kaplan–Meier analysis, which demonstrated that an increase in ApRock amplitude during DSE was associated with improved long-term survival. On the other hand, changes in LVEF during DSE did not show the same association with long-term survival.

However, Murin et al. reported that during high-dose DSE, responders exhibited a significantly higher increase in global CR compared to nonresponders (Table 1) [25]. Moreover, the responders demonstrated an average increase in LVEF of 11% ± 7%, whereas nonresponders only experienced an average increase of 2% ± 9% (*p* = 0.0007). Using a cutoff value of a 7.0% increase in LVEF during DSE, the prediction of the response to CRT resulted in a sensitivity of 79% and a specificity of 87%.

Wita et al. examined whether dyssynchrony could be used for the prediction of left ventricle reverse remodelling/CRT response (Table 1) [26]. The evaluation was conducted using two-dimensional echocardiography and TDI to assess the reverse remodelling (rLV) of the left ventricle. Furthermore, an LDDSE was performed prior to CRT. The assessment of dyssynchrony (DYS) was carried out both at rest (DYSr) and at a peak dose of LDDSE (DYSd), by measuring the difference between the time to peak systolic velocity (Ts) of the septum and the lateral wall. rLV was defined as a decrease of ≥15% of LVESV at follow-up and it was found in 67% of patients. The study showed that DYSr > 42 ms and DYSd > 59 ms had a sensitivity of 70% and 87%, specificity of 61% and 78%, and accuracy of 70% and 84%, respectively, for the prediction of rLV.

Poulidakis et al. evaluated the predictive effectiveness in identifying responders of two distinct echocardiographic strategies (Table 1) [27]. In more detail, they used LDDSE to evaluate the inotropic (CR) and inferolateral wall viability (IL), and on the other hand, a group of dyssynchrony parameters such as septal-to-posterior wall motion delay (SPWMD) by m-mode, septal-to-lateral wall delay (SLD) by TDI, interventricular mechanical delay (IVMD) by pulsed-wave Doppler for the difference in time to peak circumferential strain (TmaxCS) by speckle tracking, ApR, and septal flash (SF) by visual assessment.

**Table 1:** Studies evaluated the use of a low-dose-dobutamine stress echocardiogram in cardiac resynchronization therapy: baseline clinical characteristics of participants and parameters under examination.

Author/Year of Publication	Study Design	Clinical Characteristics	No. of Patients/ ICM (%)	Male (%)/ Mean Age	Follow-Up Period	Definition of Contractile Reserve	Definition of CRT Response	Responders (%)
**Gasparini** **2012** [21]	Multi-centre, prospective	NYHA class III–IV, optimal pharmacological therapy, LVEF ≤ 35%, LVEDD ≥ 55 mm, QRS ≥ 120 ms	221; 42.5	70; 67 ± 10	12 months	EF increase > 5%	Decrease in LVESV of ≥10%	77
**Vukajlovic** **2012** [22]	Single-centre, prospective	Nonischaemic dilated cardiomyopathy, NYHA class II–IV, LVEF < 35%, QRS > 130 ms	55; 0	83.6; 59.3 ± 10.4	28.5 ± 3.0 months	ΔEF > 14% and ΔWMSI > 0.7	EF improvement ≥ 50%, LVESD decrease <40 mm in 12 months (super = responders)	12.7 super-responders
**Mizia-Stec** **2014** [23]	Multicentre, prospective	LVEF ≤ 35%, QRS ≥ 120 ms, NYHA class III–IV, optimal medical therapy	129; 48	76; 62	6 weeks	Decrease in WMSI ≥ 0.20	Decrease in LVESV of ≥15%	81
**Stankovic** **2014** [24]	Multicentre, prospective	LVEF ≤ 35%, QRS ≥ 120 ms, NYHA class III–IV, optimal medical therapy	58; 47	77.6; 63 ± 10	41 ± 13 months	EF increase *>* 5%	Decrease in LVESV of >10%	67
**Murin** **2015** [25]	Single-centre, prospective	LVEF ≤ 35%, QRS > 120 ms, NYHA class III–IV, optimal medical therapy	52; 48	75; 62 ± 11	6 months	EF increase > 7%	Decrease in LVESV of ≥15% and/or an absolute increase of 5% in LVEF	54
**Wita** **2015** [26]	Multicentre, prospective	NYHA class III–IV, EFLV ≤ 35%, QRS ≥ 130 ms, optimal pharmacotherapy	57; 42	67; 61.9 ± 8.7	6 months	Dyssynchrony at peak LDDSE	Decrease in LVESV of >15%	68.4
**Poulidakis** **2018** [27]	Single-centre, prospective	NYHA class II–IV, maximum tolerated medical therapy, LVEF ≤ 35%, QRS > 120 ms	106; 54.7	79.2; 66.7 ± 9.8	4 years	Improvement in LVEF ≥ 20%	Decrease in LVESV of >15%	50.4

EF: ejection fraction; LVESV: left ventricular end-systolic volume; WMSI: wall motion score index; ΔEF: ejection fraction change during the dobutamine test; ΔWMSI: wall motion score index change during the dobutamine test. NYHA: New York Heart Association; LVEF: left ventricular ejection fraction.

Among the evaluated parameters, TmaxCS demonstrated the highest predictive value, with an area under the curve (AUC) of 0.835. Following that, the combination of both interventricular conduction delay and viability of the intraventricular septal wall (IL) exhibited a predictive value with an AUC of 0.799. M-mode echocardiography yielded a slightly lower predictive value with an AUC of 0.775, while the presence of either ApR or SF showed an AUC of 0.772. It is worth noting that the predictive ability of ApR and ICR was enhanced when considering late responders in addition to early responders. This suggests that including late responders in the analysis can contribute to improving the accuracy of predicting the response to CRT.

## 5. Low-Flow–Low-Gradient Aortic Stenosis with Reduced LVEF and LDDSE

In patients with reduced LVEF, a condition known as classical low-flow, low-gradient aortic stenosis (LF-LG AS) can be observed. This condition is characterized by a low transaortic flow, which leads to reductions in both the transaortic mean gradient (MG) and aortic valve area (AVA). According to guidelines, low-flow, low-gradient AS with reduced EF is defined as an aortic valve area (AVA) < 1 cm^2^, a mean gradient (MG) <40 mm Hg, EF < 50% and a stroke volume index (SVi) ≤ 35 mL/m^2^ [28]. Approximately one third of these patients have true severe AS, one third have pseudosevere AS, and the other third indeterminate AS. The management differs; aortic valve (AV) intervention is recommended in the case of true severe AS, while patients with pseudosevere AS should be treated with medical therapy [28].

LDDSE is the most common imaging method used to evaluate the severity of AS and contractile reserve and to distinguish between true severe and pseudosevere AS [1,28]. The original purpose of this test was to determine if there was CR, which refers to an increase in stroke volume (SV) of at least 20% compared to the baseline. By administering dobutamine, the LV contractility is enhanced, resulting in an increased flow and subsequently, a higher transvalvular pressure gradient [29]. This increased pressure gradient acts on the AV by causing the leaflets to bend at their base, allowing for improved flow passage. Moreover, DSE helps in estimating the projected AVA at a normal flow rate (Q) (i.e., 250 mL/s) in patients where there is still an AVA-gradient discordance. AVA projected (AVA_Proj_) is calculated based on this formula: AVA_rest_ + [(ΔAVA/ΔQ) × (250 − Q_rest_)], where AVA_rest_ and Q_rest_ are the AVA and mean transvalvular flow rate measured at rest, while the ΔAVA and ΔQ are the absolute changes in AVA and Q measured during LDDSE. With AVA_proj_ ≤ 1.0 cm^2^, AS is most likely severe [1,30]. Several observational studies examined the role of DSE in the diagnosis and prognosis of patients with LF-LG AS and reduced EF (Table 2).

Płońska-Gościniak et al. examined the usefulness of LDDSE for long-term risk assessment [31]. They found that a resting LVEF < 35% and a peak stress AVA < 0.8 cm^2^ (Table 2) (*p* = 0.003) were independent risk factors for mortality. Moreover, a small AVA at peak stress, a lack of increase in the AVA during stress, the absence of CR, and the presence of CAD were also identified as independent risk factors for death, myocardial infarctions, or pulmonary oedema during the long-term follow-up. The study further observed ongoing LV remodelling in patients with AS undergoing medical treatment, while those who underwent surgery experienced a reverse LV remodelling process and were survivors. Among surgically treated patients with true-severe AS and preserved LV contractile reserve, the perioperative mortality rate was 11%, while in patients without CR, the perioperative mortality rate was high (29%).

Annabi et al. conducted a study to evaluate the sensitivity of different criteria in distinguishing true-severe aortic stenosis from pseudosevere aortic stenosis (Table 2). They found that a DSE criterion of MG_Peak_ ≥ 40 mm Hg had limited sensitivity [25,32]. However, when they lowered the cutoff value to MG_Peak_ ≥ 35 mm Hg, the sensitivity significantly improved from 35% to 69%, and the percentage of correct classification increased from 48% to 63%. Further reducing the cutoff to 30 mm Hg did not enhance diagnostic performance. In contrast, a DSE criterion of AVA_Peak_ ≤ 1.0 cm^2^ showed a better sensitivity and a higher percentage of correct classification compared to MG_Peak_. When they adopted a cutoff value of <1.2 cm^2^ for AVA_Peak_, the sensitivity further improved from 63% to 84%. Additionally, the study demonstrated that using an indexed AVA_Proj_ ≤0.6 cm^2^/m^2^ had a sensitivity of 94%, a positive predictive value of 66%, and a percentage of correct classification of 68%.

Sato et al. aimed to assess the prognostic significance of flow reserve (FRe) in patients undergoing transcatheter aortic valve replacement (TAVR) in patients with low gradient AS and reduced EF (Table 2) [33]. The results indicated that FRe (defined as a stroke volume increase ≥20% during dobutamine stress echocardiography) did not serve as a predictor of outcomes in these patients. However, regardless of the presence or absence of FRe or the severity stratification of aortic stenosis, the study found that any form of aortic valve replacement (either surgical or transcatheter) was independently associated with improved survival.

Kim et al. investigated the association of LV global longitudinal strain (GLS) during DSE with adverse events in patients with severe LF-LG AS (Table 2). Higher values of LV GLS at peak stress and ΔGLS ≥ 2% were associated with better survival. On the contrary, the presence of CR was not associated with better outcomes [34].

In a significant study, Vamvakidou et al. showed that in patients with symptomatic LF-LG AS and reduced LVEF who underwent LDDSE, a lower stress FR was associated with a higher risk of mortality, regardless of whether they received aortic valve intervention (Table 2) [35]. Among the different criteria used to define severe AS during stress, the criterion of an AVA < 1 cm^2^ at a stress FR ≥ 210 mL/s emerged as the most accurate predictor of mortality. Stroke volume (SV) flow reserve did not demonstrate an association with mortality. Importantly, patients who met the criterion of a stress AVA < 1 cm^2^ at a stress FR ≥ 210 mL/s showed improved outcomes following aortic valve intervention, and not those who met the guideline-defined severe AS or stress MG ≥ 40 mm Hg criteria.

**Table 2:** Studies that evaluated the utility of low-dose-dobutamine stress echocardiogram in low-flow–low-gradient aortic stenosis: baseline clinical characteristics of the participants and parameters under examination.

Author/ Year	Study Design	No. of Patients/Age	Male (%)	LVEF Rest (%)	DSE Parameters	Aim	Outcome
**Płońska-Gościniak** **2013** [31]	Multicentre, prospective	39/ 59 ± 13	87.2	39 ± 8	ΕF, AVA, MG, PG, Vmax, CR (≥20% increase in LVEF)	To evaluate the long-term prognostic value of LD-DSE in patients with AS and depressed LV function	Small AVA, lack of increase in AVA, absence of CR, and the presence of CAD were independent risk factors for death
**Annabi** **2018** [32]	Multicentre, prospective	186/ 73 ± 10	78	28 ± 8	MG, AVA, SV, transvalvular flow rate, EF, AVA projected	To assess the value of MG and AVA in predicting the presence of true-severe AS and the occurrence of death in patients LF-LG AS	AVA_Proj_ better distinguished true-severe AS from pseudosevere AS and was strongly associated with mortality in patients under conservative management
**Sato** **2019** [33]	Single centre, retrospective	235/ 80	74	29	MG, Vmax, AVA, EF, GLS, CPO, flow reserve (ΔSV ≥ 20%)	To assess the prognostic impact of FR in patients with low-gradient AS	Limited ability of FR to predict outcome
**Kim** **2020** [34]	Single centre, retrospective	44/ NA	NA	31 ± 1 vs. 31.3 ± 11.6	GLS	To investigate the association of LV GLS during DSE with adverse events of patients with severe LFLG AS	The difference between GLS at rest and at peak stress had a better association with adverse events, compared to CR
**Vamvakidou** **2021** [35]	Multicentre, prospective	287/ 75 ± 10	71	31 ± 10	EF, AVA, peak and mean gradient, SV, flow rate, DVI	To assess the value of stress FR for the detection of AS severity and the prediction of mortality	Lower stress FR was an independent predictor of mortality
**Sato** **2022** [36]	Single centre, retrospective	243/ 77.6 ± 10.8	70.1	31 ± 12	MG, Vmax, AVA, EF, GLS, SV, Q, CPO, DVI, contractile reserve (ΔSV ≥ 20%)	To assess the relation between AVA, gradient, and compliance during DSE, and their impact on various markers of CR and AS severity	Flow and mean gradient increased in both the presence and absence of CR, whereas stroke volume and aortic valve area increased mainly in those with CR

AVA: Aortic valve area; FR: flow rate; LVEF: left ventricular ejection fraction; SV: stroke volume; GLS: global longitudinal strain; CR: contractile reserve.

Sato et al. evaluated retrospectively 414 DSE cases for low-gradient AS (Table 2) [37]. With the escalation of dobutamine dose, several parameters showed significant increases, including systolic blood pressure, LVEF, flow, cardiac power output, GLS magnitude, AVA, and MG (*p* < 0.05). Both flow and MG exhibited increases regardless of the presence or absence of CR, whereas SV and AVA increased primarily in those with CR alone. Notably, the aortic valve area demonstrated increases in patients with both low and high calcium scores; however, those with higher calcium scores had lower baseline area measurements. During DSE, the aortic valve area exhibited an augmentation along with an increase in the aortic valve gradient. Although a higher calcium score was associated with a lower baseline aortic valve area, the aortic valve area still increased with dobutamine, even in the presence of a high calcium score

## 6. Contractile Reserve and LDDSE

CR refers to the difference between myocardial contractility stress in comparison with rest. It is usually assessed as a change in WMSI or LVEF during stress echocardiography. Other parameters used include LV dimensions and LV global strain. CR has been shown to be associated with prognosis in patients with HF. A meta-analysis showed that the presence of CR was associated with significantly lower mortality and hospitalizations in patients with dilated cardiomyopathy [37]. In addition, patients with CR seem to respond better to pharmacological treatment and CRT [2].

## 7. Differentiating Ischaemic from Nonischaemic Cardiomyopathy and LDDSE

The sensitivity of DSE in differentiating ischaemic from nonischaemic cardiomyopathy is low. Especially in patients with severely LV dilation and extensive wall motion abnormalities, only coronary angiography could make this discrimination possible. Some of the characteristics of ischaemic heart failure during stress echocardiography are the presence of more than six akinetic segments at peak stress, less improvement in wall abnormalities, and a biphasic response [2]. Duncan et al. showed that in patients with HF, stress M-mode and pulsed-wave TDI of the lateral, septal, and posterior walls had a greater sensitivity and specificity than WMSI in discriminating an ischaemic from a nonischaemic aetiology, especially in patients with LBBB [38].

## 8. Response to B-Blockers and LDDSE

Β-blockers are part of the pharmacological treatment of HFrEF and have been shown to improve prognosis [6]. There are a few studies that investigated the role of stress echocardiography in identifying response to β-blockers. Eichorn et al. suggest that patients with CR may benefit from β-blocker therapy by improving LV function [39]. Seghatol et al. showed that the response to treatment was greater and earlier in those patients with CR [40].

## 9. Discussion

DSE imaging is particularly advantageous for patients with HFrEF and CAD due to the wide availability of echocardiography, the absence of radiation exposure, and a relatively high specificity in predicting functional recovery [41]. The accurate and noninvasive identification of viable myocardium is of great importance in patients with CAD and HFrEF. Having a reliable method to determine the viability of the heart muscle helps guide appropriate treatment strategies and predict patient outcomes [42,43]. A viability assessment during LDDSE involves the evaluation of contractile reserve. The presence of contractile reserve on an LDDSE suggests an increased likelihood of improved contractility with revascularization [44,45]. The presence of a contractile reserve in a minimum of five segments is a good predictor of LV function improvement after revascularization [46]. However, DSE’s viability testing has a lower sensitivity compared to other imaging modalities. At low doses, DSE demonstrates a sensitivity and specificity of 79% and 78%, respectively, in predicting regional functional improvement post-revascularization, with a positive predictive value of 76% and a negative predictive value of 82% [47].

Both the 2021 ESC guidelines for heart failure and cardiac resynchronization therapy do not mention the role of stress echocardiography in the evaluation of patients with heart failure who may benefit from cardiac resynchronization therapy [1,2]. However, in the EACVI/ASE recommendations on the use of stress echocardiography in nonischaemic heart disease, it is stated that stress echocardiography could help in “guiding and monitoring” response to treatment.

There are limited studies that evaluate the predictive value of LD-DSE in identifying CRT responders. LD-DSE and dyssynchrony parameters outperformed ECG criteria for CRT patient selection [27,48]. LD-DSE results along with indices such as TmaxCS, SPWMD, ApR, and SF are simple and reliable predictors of CRT response, not only within 6 months, but of the late response as well [27]. Combining an assessment of the contractile reserve with dyssynchrony indices can enhance the sensitivity and specificity of this diagnostic test [49,50]. However, it is important to note that the decision to implant CRT should not be based solely on the presence or absence of contractile reserve. Further investigation through future trials is needed.

LDDSE is a valuable tool for classifying AS and guiding decision-making. Parameters such as mean gradient, AVA, projected AVA, SV, flow rate, and GLS can help identify true severe AS, estimate prognosis, and determine appropriate management for each patient [51]. The conventional criteria used to define true severe AS, such as mean gradient and AVA, often lead to the misclassification of AS severity due to the presence of low-flow states and an AVA–mean gradient discordance [52]. According to studies, AVA_Proj_ can be useful in distinguishing true severe AS from pseudosevere AS and is also a good predictor of mortality [32]. Additionally, a lower stress flow rate, rather than the absence of flow reserve, has been identified as an independent predictor of mortality in patients with LF-LG AS and reduced EF [35]. The flow rate also provides indications of outcomes following aortic valve intervention [35,52]. However, the results of these observational studies require further validation through randomized clinical trials.

Finally, stress B-lines observed during echocardiography can provide information about the hemodynamic profile of patients with heart failure. It can be helpful in guiding management decisions by identifying patients at risk of decompensation at an early stage [53]. Combining stress B-lines with clinical parameters and cardiac natriuretic peptides can provide valuable prognostic information [37,38,39].

The use of a single individual cardiac stress imaging test has a significant economic and public health impact. The Stress echo (SE) 2020 is a multicentre, prospective study started in 2016 which resulted in the development and validation of the ABCDE protocol for functional imaging [54,55]. The five steps included in the protocol are: step A, the assessment of epicardial flow by regional wall abnormalities; step B, the assessment of diastolic reserve and pulmonary congestion through B-lines by lung ultrasound; step C, left ventricular contractile reserve; step D, coronary flow velocity reserve; step E: the evaluation of chronotropic reserve through heart rate by ECG [54]. The ABCDE+ protocol includes additional steps: the measurement of gradients and regurgitant flows, the evaluation of left atrium volume and function, as well as pulmonary and left ventricular pressures, and the assessment of right ventricular function [55]. This comprehensive approach covers a broad spectrum of clinical conditions and phenotypes: coronary artery disease, hypertrophic cardiomyopathy heart failure with preserved ejection fraction (HFpEF), and valvular and congenital disease. Moreover, it could be a valuable tool to evaluate patients after chest radiotherapy or COVID-19 infection [55]. ABCDE and ABCDE+ protocols can be tailored to the individual patient according to clinical needs.

## 10. Conclusions

Stress echocardiography offers several key benefits. It is a straightforward, cost-effective, easily accessible, and radiation-free diagnostic test. When contractile reserve is observed during LDDSE, it indicates a higher probability of enhanced contractility following revascularization. In patients with heart failure with reduced ejection fraction, LDDSE serves as a valuable tool for both categorizing aortic stenosis and aiding in decision-making processes. The combination of LDDSE and dyssynchrony parameters demonstrates superior performance compared to ECG criteria in selecting patients for cardiac resynchronization therapy.

## Data Availability

Not applicable.
